# Postprandial Glycaemic, Hormonal and Satiety Responses to Rice and Kiwifruit Preloads in Chinese Adults: A Randomised Controlled Crossover Trial

**DOI:** 10.3390/nu10081110

**Published:** 2018-08-17

**Authors:** Alex Lubransky, John Monro, Suman Mishra, Hui Yu, Jillian J. Haszard, Bernard J. Venn

**Affiliations:** 1 Department of Human Nutrition, University of Otago, P.O. Box 56, Dunedin 9054, New Zealand; alex.lubransky@gmail.com (A.L.); yuev5129@student.otago.ac.nz (H.Y.); jill.haszard@otago.ac.nz (J.J.H.); bernard.venn@otago.ac.nz (B.J.V.); 2 New Zealand Institute for Plant & Food Research Ltd., Private Bag 11600, Palmerston North 4442, New Zealand; Suman.Mishra@plantandfood.co.nz

**Keywords:** fructose, glycaemia, insulinaemia, preload, kiwifruit, fruit

## Abstract

Controlling postprandial glycaemia helps to prevent and manage non-communicable diseases. One strategy in controlling glycaemia may be to consume meals in two parts; a preload, followed by the remainder of the meal. Our aim was to test preloading a rice meal given for breakfast and lunch on different days, either by splitting the meal (rice preload followed by rice meal) or by using kiwifruit as a preload compared with consuming the rice meal in one sitting. Primary outcomes were glycaemic and insulinaemic responses with secondary outcomes of other hormonal responses, subjective satiety, and subsequent energy intake. Following breakfast, postprandial glycaemic peak concentration was 0.9 (95% CI: 0.2, 1.6) mmol/L lower for the kiwifruit preload compared with the rice meal eaten in one sitting. Following lunch, glycaemic peak concentrations were 1.0 (0.7, 1.4) and 1.1 (0.5, 1.7) mmol/L lower for the rice-split and kiwifruit preload compared with the rice meal alone, respectively. Postprandial insulinaemia area-under-the-curve was 1385 (87, 2684) mU/L·min less for the kiwifruit preload compared with the rice-split. There were no differences among treatments for subsequent energy intake. Meal splitting is useful for lowering postprandial glycaemia, and replacing part of a meal with kiwifruit may help with insulin efficiency without detriment to subsequent energy intake.

## 1. Introduction

Given the worldwide prevalence of prediabetes and diabetes mellitus, there is interest in managing postprandial blood glucose concentration [1]. One strategy has been to advise people to eat fatty or high protein foods before eating the carbohydrate portion of a meal [2]. The rationale for this is that a fat preload delays gastric emptying and dampens glycaemia [3,4], and a protein preload reduces glycaemia by stimulating insulin [5,6]. However, the type of fatty acids ingested with carbohydrate has health implications, as diets enriched with *trans*- and saturated fatty acids increased postprandial insulinaemia relative to a baseline diet containing an equivalent amount of fat, a metabolic state indicative of post-meal insulin resistance [7]. Although co-ingestion of fat and carbohydrate may effectively reduce glycaemia, consumer knowledge about the types of fatty acids is poor, and the consumer perception of dietary fat as being unhealthy may preclude widespread uptake of this strategy [8]. While carbohydrate co-ingested with 25 g protein contained in beef, turkey, gelatin, egg white, cottage cheese, fish, or soy foods has been found to reduce postprandial glycaemia, in each case, this was accompanied by a 2- to 4-fold increase in postprandial insulin [9]. Increases in postmeal insulin may be undesirable, as postprandial hyperinsulinemia has been found to be independently associated with coronary artery disease risk among women without diabetes [10]. For people with type 2 diabetes, the use of an insulin-stimulating drug has been associated with adverse cardiovascular outcomes [11] and there is evidence to suggest that protein restriction may slow the progression of diabetic kidney disease [12]. Thus, the use of protein to increase postprandial insulin may be contraindicated in people with and without diabetes [10,12].

Other options for controlling glycaemia include manipulating the frequency of meals [13]. It has been found that compared with three larger meals, smaller meals consumed throughout the day reduced glycaemic and insulinaemic fluctuations and improved postprandial insulin sensitivity [14,15]. The inclusion of fruit (raisins) in a starchy meal (oats) had no deleterious effect on postprandial glycaemia and insulinaemia in people with type 2 diabetes [16]. The addition or introduction of fruit into diets may be useful because whole-fruit consumption has been associated with a lower risk of type 2 diabetes [17]. Fruit consumption is consistent with dietary advice, as given by health authorities across Europe and North America [18,19,20]. Despite these recommendations, the inclusion of sugars in the diet has attracted negative publicity, with fructose being of particular concern [21]. Thus, an apparent contradiction arises in that fruit (containing fructose) is healthy, while the metabolism of fructose is suggestive of potentially harmful effects [22]. This has led to commentary that fructose is only a problem when consumed in excess from processed foods as a component of sucrose or high fructose corn syrup, and that fruit can be tolerated by humans in large quantities [23].

These observations led us to speculate that splitting a predominantly carbohydrate-containing meal (white rice) might have glycaemic benefit over consuming the rice in one sitting; in effect, creating a carbohydrate preload of rice followed by the remainder of the rice meal. An expansion of this concept involved the replacement of the rice preload on an equi-carbohydrate basis with kiwifruit. The purpose of replacing rice starch with kiwifruit sugars derives from the fructose component of kiwifruit, as fructose is less glycaemic and less insulinaemic compared with glucose [24]. Additional benefits from substituting kiwifruit for cereal may be obtained from kiwifruit properties that retard physical processes of carbohydrate digestion in vitro [25]. In vivo, it has been found that kiwifruit co-ingested with cereal reduces postprandial glycaemic responses in comparison to the cereal ingested alone, by more than could be explained by simple fructose substitution of starch [26]. Neither the acute effects of meal-splitting nor kiwifruit-for-starch preload substitution on postprandial glycaemic, hormonal, and satiety responses have been tested. From previously published work, an appropriate time interval between the preload and the remainder of the meal is 30 min, a time lapse that has been recommended to investigate gastrointestinal and satiety effects [27]. Indeed, the compensation of energy intake compared with no preload was most precise when a 30 min interval was used, compared with longer time intervals [28]. A priori, the study was designed for Chinese participants, as postprandial glycaemia has been found to be higher in Chinese people compared with people of European descent [29].

Hence, the primary aims of this research were to establish whether meal-splitting and incorporating kiwifruit into a starchy meal by carbohydrate exchange would be beneficial in terms of postprandial glycaemic and insulinaemic responses compared to a single rice meal, with secondary aims of measuring other postprandially affected hormones and monitoring the satiety of the meals in healthy adults. In practice, people might split a meal or substitute a preload at different meals; thus, the experiment was to be carried out both at breakfast time and at lunch time on separate days, with a two-day washout.

## 2. Materials and Methods

### 2.1. Ethics and Design

The study was approved by the University of Otago Human Ethics Committee (Health), reference H16/066, dated 9th June, 2016. This study has been registered with the Australian and New Zealand Clinical Trials Registry ACTRN12616000771459.

**Figure 1:**
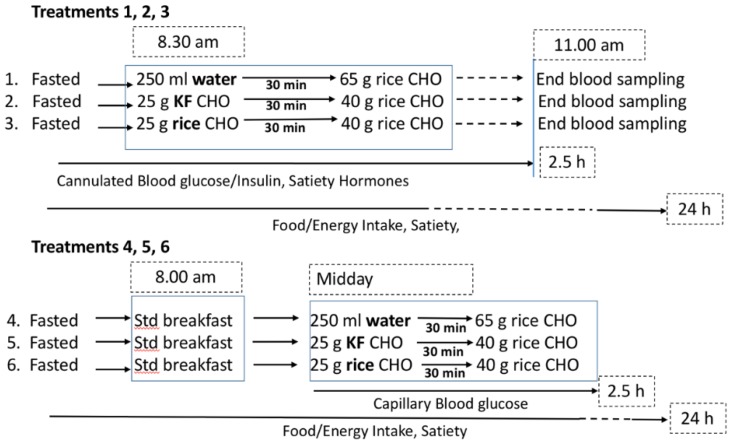
Summary of experimental design. KF = kiwifruit; CHO = carbohydrate, Std breakfast = standard breakfast.

The trial used an unblinded, randomised, repeated measures cross-over design with 30 participants (Figure 1). Participants were allocated randomly to undertake the breakfast or lunch testing first. The treatment order was then randomised separately for breakfast and lunch testing.

### 2.2. Meal Components

Kiwifruit (*Actinidia chinensis* var. *chinensis* ‘Zesy002’, marketed as Zespri® SunGold Kiwifruit) of export quality were provided in a ready-to-eat state of ripeness by Zespri International Limited, Tauranga, New Zealand. The carbohydrate content of the kiwifruit was determined by standard colourimetric methods from the fructose and glucose content after preliminary amyloglucosidase and invertase hydrolysis. Zespri International Ltd provided the nutritional composition of kiwifruit (Table 1) except that the available carbohydrate content was measured directly on the fruit used in the trial at the Plant and Food Research Laboratories by extraction with 80% ethanol, hydrolyzing an aliquot with invertase, and measuring total reducing sugars and fructose colorimetrically (Glucose:fructose 0.94:1) Starch was not measured, as previous analyses have shown that it constitutes only about 1% of available carbohydrate in ripe SunGold kiwifruit [30].

The rice meal consisted of a rice porridge served in a chicken-flavoured broth as the traditional Asian food known as congee, for which all ingredients were obtained from a local supermarket. The congee was prepared in the metabolic kitchen of the Human Nutrition department at the University of Otago in the hour prior to participants arriving for testing (between 0700–0800 h for breakfast; between 1100–1200 h for lunch). Jasmine rice (SunRice, Sydney, Australia) was cooked in a rice cooker with water and chicken stock until the desired consistency was reached. Boiled shredded chicken breast, fresh chopped spring onion, fried shallots, and sesame oil were added to the top of the cooked rice before it was served.

On lunch testing days, a standard breakfast consisting of two steamed pork buns and a cup of Chinese green tea was provided to participants. The compositions of the congee and steamed bun were determined using standard food analytical methods by Cawthron Analytical Services, Nelson, New Zealand (Table 1).

The weight of congee provided in a complete meal was 468 g. The split congee meal was given as 180 g preload followed thirty minutes later by a 288 g serving size. The congee given as a split meal (rice preload) or as eaten in one sitting (water preload) had an energy content of 1797 kJ and a carbohydrate content of 65 g. The carbohydrate content of the preload (both rice and kiwifruit) was 25 g. The overall energy content of the kiwifruit preload and congee meal was 1583 kJ.

### 2.3. Participants

The inclusion criteria were adults aged 18–75 years, self-identified as being ethnically Chinese. Recruitment was by posters and flyers placed around the campus of the University of Otago, New Zealand, and at adjacent workplaces. Respondents were assessed according to the inclusion and exclusion criteria for the study. Exclusion criteria were inability to speak English, self-reported disease of the digestive system (coeliac, Crohn’s diseases), having had gastrointestinal surgical procedures, an allergy to kiwifruit, and pregnancy. Diagnosis of other chronic diseases (diabetes mellitus, cardiovascular disease) did not exclude participation. Respondents that were potentially eligible to take part were provided with an information sheet to take away and consider. People willing to participate were booked in for an initial visit, during which a screening questionnaire was filled in, eligibility criteria were rechecked, and people were given an opportunity to ask questions. When satisfied, participants signed a consent form, filled out a personal information questionnaire, and had their height and weight measured. Reimbursement of $150 in supermarket vouchers was given for a complete set of six tests (or pro rata if participants withdrew).

### 2.4. Blood Sampling

Participants were asked to fast overnight and to avoid any strenuous exercise before testing. Blood samples were taken at baseline and at 15, 30, 45, 60, 75, 90, 120, and 150 min thereafter.

#### 2.4.1. Breakfast Test

For the breakfast tests, blood samples were withdrawn into K EDTA-treated blood collection tubes by a cannula inserted into a vein in the cubital fossa by a research nurse. An initial (baseline) sample was taken before ingesting any food, after which the participants consumed a pre-randomised preload within 10 min. Further blood was drawn at 15 and 30 min. At 30 min, participants were given breakfast and asked to consume this within 15 min. Blood samples were taken at 45, 60, 75, 90, 120, and 150 min following baseline, making a total of nine blood draws. At each time point, participants filled out a satiety questionnaire that involved making marks on four visual analogue scales to indicate that person’s degree of satiety. The questionnaire was used on each of the six days of testing (Figure 1). The questions and the extremes (in brackets) were:

How hungry do you feel at this moment? (Not at all hungry ------ Extremely hungry)
How full does your stomach feel at this moment? (Not at all full ------ Extremely full)
How strong is your desire to eat at this moment? (Very weak ------ Very strong)
How much food do you think you could eat at this moment? (Nothing at all ------ A very large amount).

All questions were combined into a total appetite scale by taking the mean of the four questions, after reverse scoring the “How full does your stomach feel at this moment?” item. The total appetite score ranged from a possible 0 to 10 cm, with 10 indicating a high level of hunger.

#### 2.4.2. Lunch Test

On lunch test days, participants attended the clinic at 0800 h to eat a standard breakfast, which consisted of two steamed pork buns and hot green tea; participants were then free to leave the clinic but were requested to return four hours later without meanwhile consuming any other food. The steamed buns were purchased from a local Asian supermarket in frozen packs of 10. The buns were stored frozen and steamed until hot before serving to participants. For lunch, the same rice preparation and preload procedure as the breakfast test was used, but blood glucose was measured as capillary blood glucose collected via finger prick rather than by cannula. The purpose of giving a standard breakfast was to standardise the meal prior to lunch to reduce variation in a possible ‘second-meal effect’, a phenomenon by which the glycaemic response to a meal is influenced by the preceding meal.

### 2.5. Blood Analysis

Blood glucose in plasma samples from the cannula draws at breakfast were measured using a Cobas c311 analyser (Roche, Germany). At lunch, capillary blood glucose concentration was measured using a HemoCue (Ängelholm, Sweden) blood glucose analyser. The glycaemic data were compared using measures of incremental area under the blood glucose response curve (iAUC; mmol/L·min) and for glucose, peak height (BGRmax; mmol/L·min).

Blood hormones (insulin, ghrelin, glucagon, and GLP-1) were determined by Multiplex Elisa analysis (Thermo Fisher Scientific, Waltham, MA). The data are expressed as mean concentration over the 150 min period following commencement of the breakfast test.

### 2.6. Energy Intake

Participants were given a set of electronic kitchen scales and instructed to weigh all of their food and beverage intake consumed for the remainder of the day following the tests. Participants recorded the name, brand, and weight of the food or beverage, and the time of eating. For homemade food, participants were asked to record each raw ingredient and preparation method. The food diary entries were entered into a nutrient analysis software program (Kai-calculator version v1.15s, Department of Human Nutrition, University of Otago) that sources its nutritional information from the New Zealand food composition database [30].

### 2.7. Statistical Methods

Based on data derived from a pilot study, 25 participants would be sufficient to detect a clinically significant difference in blood glucose response of 25% (approximately the difference between a low and a high glycaemic index food) using a significance level (α) of 0.05 and with a power of 0.9. We over-recruited (*n* = 30) to allow for dropouts. Stata 15.1 (StataCorp, College Station, TX, USA) was used for all statistical analyses. Differences in treatments were determined using mixed effects regression models with participant id as a random effect, robust standard errors, and adjusted for randomised order. Mean differences, 95% confidence intervals, and p-values were calculated.

## 3. Results

**Figure 2:**
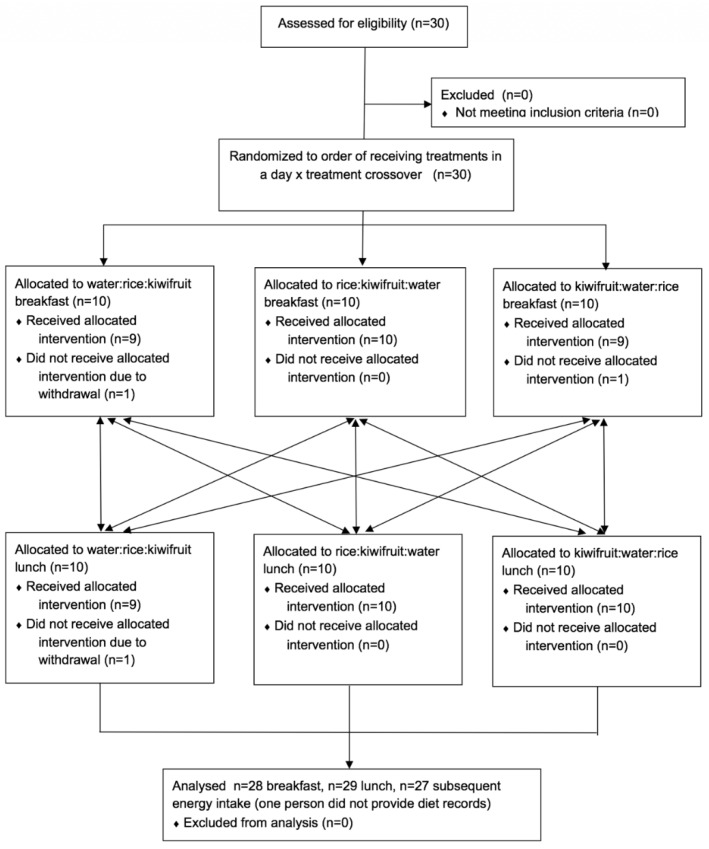
Participant flowchart.

Thirty healthy Chinese participants, 25 female and 5 male, aged 19–41 years with a mean (SD) body mass index of 21.8 (3.8) kg/m^2^, were enrolled in the study. Twenty-eight participants completed all three treatment arms at breakfast, and 29 completed at lunch. One female completed the lunch treatments only, and another female withdrew without providing any data. Participants were randomised to the order in which they received the treatments, as shown in Figure 2.

### 3.1. Blood Glucose

**Figure 3:**
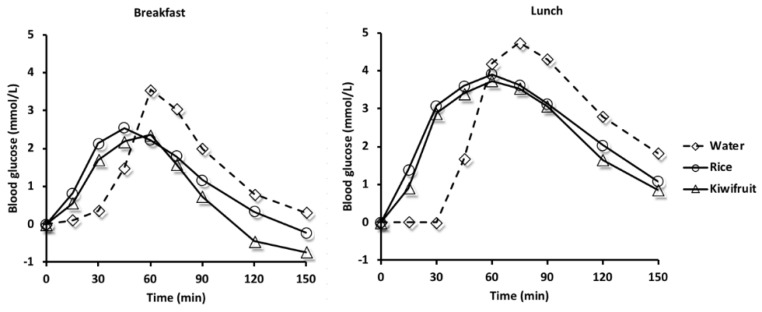
Mean incremental change in blood glucose concentrations at breakfast (*n* = 28) and at lunch (*n* = 29) given on different days from baseline (time 0), at which time preloads of water, rice, or kiwifruit were followed at 30 min by the remainder of the rice meal.

The mean (SD) baseline blood glucose concentrations at breakfast were 5.2 (0.7), 5.2 (0.5), and 5.3 (0.6) mmol/L, and at lunch, 4.6 (0.5), 4.6 (0.6), and 4.4 (0.6) mmol/L for the water, rice, and kiwifruit treatments, respectively. The mean incremental rise in blood glucose concentration over time is plotted in Figure 3.

Water given as a preload half an hour before the rice meals resulted in no rise in blood glucose concentration whereas when rice and kiwifruit were given as preloads, there was a continuous rise in blood glucose throughout the first 45–60 min. Although the rice meals were identical in nutrient content, the postprandial rise in capillary blood glucose response (iAUC) was considerably larger at lunch compared with venous blood collected at breakfast (*p* < 0.05 for all treatments).

Incremental areas-under-the-glucose-curves (iAUC), peak glucose concentration, time to peak and comparisons of these factors among treatments are given in Table 2 for both breakfast and lunch. Peak capillary blood glucose concentration after lunch generally occurred at the 75 and 90 min timepoints and exceeded 10 mmol/L in nine, three, and three participants, following the water preload, split meal and kiwifruit preload, respectively.

The treatments comprised a preload of water, rice or kiwifruit ingested 30 min before the same rice meal eaten for breakfast on three days and for lunch on another three days: *n* = 28 at breakfast; *n* = 29 at lunch.

At breakfast, the kiwifruit preload resulted in a smaller glucose iAUC compared with the water preload; there was no difference in glucose iAUC among treatments at lunch. Peak glucose was lower following the kiwifruit compared with the water preload at breakfast, and both the rice and the kiwifruit preloads resulted in lower peak glucose at lunch compared with water. Time to peak was shorter at breakfast and at lunch for the rice and kiwifruit preloads, compared with water. There was no difference for any of these factors between the rice and kiwifruit treatments.

### 3.2. Hormones

**Figure 4:**
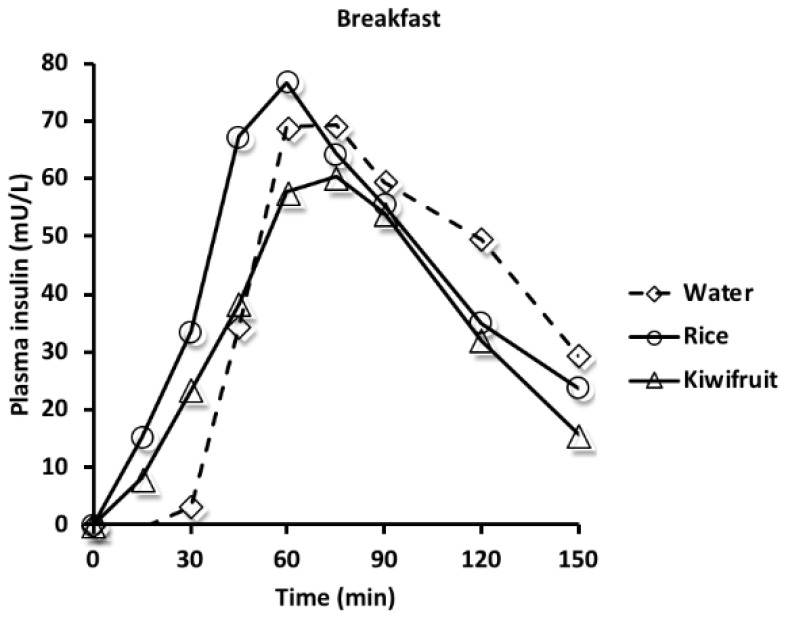
Mean incremental change in plasma insulin concentrations at breakfast from baseline (time 0) at which time preloads of water, rice, or kiwifruit were followed at 30 min by the remainder of the rice meal.

The mean (SD) baseline plasma insulin concentrations at breakfast were 7.6 (5.8), 6.8 (6.3), and 6.4 (4.5) mU/L for the water, rice, and kiwifruit treatments, respectively. The mean incremental rise in plasma insulin concentration over time is plotted in Figure 4. 

Water given as a preload half an hour before the rice meals resulted in a minimal rise in plasma insulin concentration, whereas when rice and kiwifruit were given as preloads, there was a continuous rise in plasma insulin throughout the first 60 min. Postprandial incremental areas-under-the-insulin-curves (iAUC) and mean concentration of plasma ghrelin, glucagon, and GLP-1 over 150 min after breakfast are given in Table 3.

The kiwifruit preload resulted in a smaller plasma insulin iAUC compared with the rice preload. There was no difference among treatments for mean plasma ghrelin concentration. The mean plasma glucagon concentration was higher for the kiwifruit compared with the rice preload; and for GLP-1, the concentration was lower for the rice compared with the water preload.

### 3.3. Satiety and Subsequent Energy Intake

Subjective appetite for a duration of 150 min after breakfast and lunch, and subsequent energy intake throughout the remainder of the days are given in Table 4. One male participant did not provide diet records following the tests.

Following breakfast, a greater appetite was reported after the kiwifruit compared with the water and the rice preloads. Following lunch, a smaller appetite was reported after the rice compared with the water preload; and a greater appetite after the kiwifruit compared with the rice preloads. There was no difference among treatments in the subsequent energy intake for the rest of the day following breakfast or lunch.

## 4. Discussion

The main findings of the study are that a predominantly carbohydrate preload given 30 min before the remainder of the meal changes the shape of the glycaemic response curve by suppressing the peak concentration. This effect was found after breakfast and after lunch for both the rice and kiwifruit preloads, compared with the water preload treatment. Consistent with the literature, postprandially venous blood has lower glucose concentrations than capillary blood [31]. However, our main outcome was a comparison among treatments and the pattern of response was similar between breakfast and lunch, despite the different blood pools sampled (venous at breakfast and capillary at lunch).

Controlling peak glucose may be important, as postprandial glycaemic peaks have been associated with higher glycated haemoglobin concentrations and with thicker carotid intima-media thickness in people with type 2 diabetes [32]. Due to evidence of increased cardiovascular risk, maintaining a peak postprandial capillary blood glucose concentration below a threshold of 10 mmol/L is a recommendation of the American Diabetes Association [33]. Three participants exceeded 10 mmol/L following the split meal and the kiwifruit preload meal, compared with nine following the meal eaten in one sitting, a finding consistent with a glycaemic benefit of spreading the time-course of consuming a meal.

This study has shown that consuming fruit, recommended for multiple health benefits, need not cause high postprandial glycaemic responses due to fruit sugars. Reasons for this are firstly, that fructose is intrinsically less glycaemic than cooked starch in most cereal products [34]. This quality is consistent with the concept that when used in an equicarbohydrate exchange format, as in the present study, glycaemic response is likely to be reduced. Secondly, increasing meal frequency from three to six per day spreads the ingestion period, and using this approach, it has been found that fluctuations in postprandial glycaemic and insulinaemic responses are evened out [15], and that postprandial insulin sensitivity is improved [14]. Splitting the meal into a preload and a main meal without increasing carbohydrate, as in the present study, is an example of an extended ingestion period in which realistic intakes of preload and main meal were used. In the present study, 25 g of available carbohydrate in a total of 65 g carbohydrate was ingested in the preload, that is, 38% of the carbohydrate. However, as the kiwifruit sugars yield about 50% glucose and 50% fructose, the fructose alone would have substituted about 19% of the rice starch, so the contribution of substitution by fructose to any overall effects could be expected to be modest. Nevertheless, the study has shown that consuming kiwifruit as a preload did reduce glycaemic response—at breakfast, the kiwifruit preload reduced iAUC by about 30% compared with 12% for the rice preload, possibly reflecting the combined effect of fructose substitution and the gut-level action of other kiwifruit components at digestion, as found previously [26]. The results indicate the safety of consuming fruit in a carbohydrate exchange format, particularly when carbohydrate loading is attenuated by preloading.

Drugs have been developed to target postprandial glycaemia, and there are dietary strategies that have been tested, including adding protein and fat to carbohydrate, limiting carbohydrate intake, and reducing the carbohydrate load by making food choices based on the glycaemic index. Suppression of peak glucose has been reported previously with protein preloads [6,35]. The mechanism by which a protein preload has a moderating effect on glycaemia is by stimulating a greater insulin response [6]. This strategy for reducing glycaemia may not be ideal, as total and animal protein intakes have been associated with increased risk of developing type 2 diabetes [36]. Additionally, drugs designed to stimulate insulin have been associated with major adverse events in people with type 2 diabetes [11,37]. Another method used to reduce postprandial glycaemia is to limit carbohydrate intake by increasing the fat content of the diet. When applied for one or two days, this strategy has been found to be effective at reducing postprandial glycaemia but unless this dietary pattern is maintained, a return to more usual carbohydrate intakes results in exaggerated postprandial glucose excursions [38,39]. When low carbohydrate diets have been followed for up to three months, some improvement in glycaemic control in people with type 2 diabetes has been found [40,41], but it has been difficult to show maintenance of glycaemic benefit in the longer-term [42,43,44]. The concept of the glycaemic index has been used to reduce postprandial glycaemia. In this scenario, the macronutrient composition of the diet can be maintained whilst choosing foods on the basis of imparting low glycaemic responses [45]. Peak glucose concentration has been reduced when low, compared with high glycaemic index foods, have been chosen [46]. This strategy does rely on people being able to choose appropriate foods, and when applied over a number of weeks or months, has not always shown glycaemic benefit [47,48,49].

Co-ingestion of fat and carbohydrate has been found to reduce postprandial glycaemia, but this may not be a healthy strategy, as fat is calorically more dense than protein and carbohydrate; triglycerides and insulin were found to be raised by the combination of macronutrients indicative of fat intake, potentiating insulin secretion to the detriment of insulin sensitivity [50]. Similarly, the ability of ingested protein to potentiate insulin secretion under euglycaemic clamp conditions is indicative of peripheral insulin resistance [51]. The strength of a carbohydrate preload approach to lower peak glucose is that insulin concentrations were not increased compared with a single meal. Additionally, usual foods can be consumed, albeit with a 30-min time gap as in our study, between the preload and the remainder of the meal. Peak glucose was reduced when the rice meal was split and when kiwifruit was given as a preload. An advantage of using kiwifruit compared with the split rice meal was a lower insulin demand with the kiwifruit preload. Despite the lower insulin demand, the blood glucose concentration fell below baseline with the kiwifruit treatment, perhaps indicating a higher mean glucagon concentration to correct for this undershoot. An undershoot might have been expected, given that this is a characteristic of consuming foods containing fructose, including fruit [52]. However, the lower peak glucose, together with the reduced insulin demand, are potentially beneficial, as postprandial glycaemia and resultant insulin responses are positively associated with postprandial arterial stiffness [53].

The rice and kiwifruit preload meals were associated with a greater appetite score over the postprandial period compared with when the rice meal was eaten in one sitting. However, despite this subjective difference, there was no difference among treatments in subsequent energy intake throughout the test days. Our data are consistent with the use of water and fruit preloads in a weight loss setting; the preload concept being equally effective among treatments [54]. However, an advantage of using kiwifruit as a preload over the rice and water preloads was a lower energy content of the overall meal compared with using rice alone. The strategy of providing a low energy dense fruit as a preload to a starchy meal appears to be beneficial in these Chinese participants.

Although potentially beneficial effects on glycaemia and insulinaemia have been found in an acute setting, a limitation of the work described here is whether people would be willing to habitually adopt the concept of spreading a meal over a longer duration. Some people may not have the time, some may, and others might be able to reorganise their schedule to fit. It may be useful to test shorter time periods between preloading and the remainder of a meal. Other considerations would be the types of foods and mealtimes that people would be willing to apply the concept to. Splitting a rice meal, as was done here would require keeping the rice warm over an extended period, and this may be unacceptable to some people. The use of kiwifruit as a preload has the advantage over splitting a heated meal in that the fruit and the main meal are separate items. Kiwifruit has qualities that may make it particularly suitable for use in this way [25], but kiwifruit may be unobtainable throughout the year, and it would be informative in future research to compare the metabolic potential of other fruits. Our work is also limited in its demographic generalisability. Our participants were Chinese because postprandial glycaemia has been found to be higher in Chinese people compared with people of European descent [29,55]. It would be of interest to prepare meals containing other carbohydrate-rich foods to test the generalisability of findings among foods and other population groups, including by age, ethnicity and glucose tolerance.

## 5. Conclusions

Splitting the congee meal (rice preload) or using kiwifruit as a preload resulted in lower glycaemic responses compared with the congee eaten in one sitting, suggestive of a glycaemic advantage. Additional benefits of the kiwifruit preload were lower insulinaemia compared with the split meal and a lower energy content of the kiwifruit/congee combination compared with the congee alone, with no difference among meals in subsequent energy intake.

## Figures and Tables

**Table 1:** Composition of foods consumed.

Component	Kiwifruit	Rice Porridge (Congee)	Steamed Bun
Energy (kJ/100 g)	238	384	911
Protein (g/100 g)	1.02	4.1	6.6
Fat (g/100 g)	0.28	2.1	3.2
Carbohydrate (g/100 g)	13.1	13.9	40
Moisture (g/100 g)	82.4	79.7	49.1
Ash (g/100 g)	0.47	0.2	1.1

**Table 2:** Postprandial blood glucose responses to treatments (preloads and meals combined) over 150 min.

Blood Glucose	Mean (SD)			Mean Difference (95% CI)		
	Water Preload + Rice	Rice Preload + Rice	Kiwifruit Preload + Rice	Rice vs. Water	Kiwifruit vs. Water	Kiwifruit vs. Rice
Breakfast iAUC ^1^ (mmol/L·min)	218 (171)	191 (125)	153 (93)	−26 (−82, 30) *p* = 0.357	−65 (−129, −1) *p* = 0.047	−39 (−80, 2) *p* = 0.063
Lunch iAUC (mmol/L·min)	365 (185)	371 (156)	336 (146)	6 (−38, 50) *p* = 0.788	−29 (−76, 19) *p* = 0.237	−35 (−82, 12) *p* = 0.147
Breakfast peak (mmol/L)	9.1 (2.0)	8.4 (1.4)	8.1 (1.1)	−0.7 (−1.4, 0.1) *p* = 0.107	−0.9 (−1.6, −0.2) *p* = 0.010	−0.3 (0.2, −0.8) *p* = 0.264
Lunch peak (mmol/L)	9.7 (1.6)	8.7 (1.1)	8.6 (1.5)	−1.0 (−1.4, −0.7) *p* < 0.001	−1.1 (−1.7, −0.5) *p* < 0.001	−0.1 (−0.5, 0.4) *p* = 0.761

^1^ iAUC = incremental area-under-the-curve.

**Table 3:** Mean postprandial hormonal responses to breakfast treatments (preloads and meals combined) over 150 min (*n* = 28).

Hormone	Mean (SD)			Mean Difference (95% CI)		
	Water Preload + Rice	Rice Preload + Rice	Kiwifruit Preload + Rice	Rice vs. Water	Kiwifruit vs. Water	Kiwifruit vs. Rice
Insulin iAUC ^1^ (mU/L·min)	5962 (2858)	6552 (3437)	5167 (2779)	498 (−688, 1685) *p* = 0.296	−887 (−1894, 119) *p* = 0.152	−1385 (−2684, −87) *p* = 0.036
Ghrelin (pg/mL)	32.9 (25.6)	35.1 (34.7)	32.3 (21.2)	2.2 (−2.5, 6.8) *p* = 0.359	−0.6 (−5.3, 4.0) *p* = 0.787	−2.8 (−7.4, 1.8) *p* = 0.235
Glucagon (pg/ml)	25.3 (10.8)	24.3 (10.8)	26.9 (14.9)	−1.0 (−3.0, 1.1) *p* = 0.353	1.6 (−0.5, 3.6) *p* = 0.134	2.5 (0.5, 4.6) *p* = 0.015
GLP-1 (pg/mL)	149 (44)	137 (47)	142 (41)	−12 (−18, −5) *p* < 0.001	−6 (−13, 0.3) *p* = 0.063	5 (−1, 12) *p* = 0.100

^1^ iAUC = incremental area-under-the-curve.

**Table 4:** Appetite responses via visual analogue scales, and subsequent energy intake following treatments (note: the breakfast and the lunch tests were on different days).

Satiety and Energy Intake	Mean (SD)			Mean Difference (95% CI)		
	Water Preload + Rice	Rice Preload + Rice	Kiwifruit Preload + Rice	Rice vs. Water	Kiwifruit vs. Water	Kiwifruit vs. Rice
Appetite after breakfast cm·min	593 (177)	605 (226)	687 (218)	12 (−39, 63) *p* = 0.650	94 (39, 149) *p* = 0.001	82 (26, 138) *p* = 0.004
Appetite after lunch cm·min	690 (229)	631 (242)	724 (261)	−59 (−103, −15) *p* = 0.009	34 (−19, 86) *p* = 0.208	93 (28, 157) *p* = 0.005
Energy intake after breakfast (MJ)	6.08 (2.02)	5.99 (2.44)	6.08 (2.34)	−0.09 (−0.94, 0.76) *p* = 0.836	0.00 (−0.88, 0.89) *p* = 0.995	0.09 (−0.72, 0.90) *p* = 0.823
Energy intake after lunch (MJ)	4.22 (2.20)	4.41 (1.87)	4.32 (1.54)	0.19 (−0.58, 0.96) *p* = 0.629	0.10 (−0.63, 0.83) *p* = 0.788	−0.09 (−0.74, 0.56) *p* = 0.786

Appetite scale combined all four items, with the ‘how full do you feel’ item reversed. A higher score indicates a greater appetite. Appetite *n* = 28 at breakfast; *n* = 29 at lunch; Energy intake *n* = 27.
